# Malaysian adolescents’ exposure to secondhand smoke in the car of their parents/guardians: A nationwide cross-sectional school-based study

**DOI:** 10.18332/tid/122586

**Published:** 2020-06-12

**Authors:** Kuang H. Lim, Hui L. Lim, Sumarni M. Ghazali, Chee C. Kee, Chien H. Teh, Balvinder S. Gill, Mohd Z. Taib, Pei P. Heng, Jia H. Lim

**Affiliations:** 1 Special Resource Centre, Institute for Medical Research, Kuala Lumpur, Malaysia; 2 Oncology Department, Hospital Sultan Ismail, Johor Bahru, Malaysia; 3 Sector for Biostatistics and Data Repository, National Institutes of Health, Shah Alam, Malaysia; 4 School of Pharmacy, Monash University Malaysia, Bandar Sunway, Malaysia

**Keywords:** school-going adolescent, parent/guardian transport, secondhand smoke, smoking status

## Abstract

**INTRODUCTION:**

We investigated the prevalence of children’s exposure to secondhand smoke (SHS) in the car of their parents/guardians and the associated factors.

**METHODS:**

A self-administered validated questionnaire was used to obtain data from the nationally representative samples of school-going adolescents aged 11–19 years in Malaysia. Prevalence rates were computed and chi-squared tests and multiple logistic regression were conducted.

**RESULTS:**

Of the participants, 23.3% reported exposure to SHS at least once in the car of their parents/guardians during the last 7 days before the survey. The prevalence and likelihood of SHS exposure were significantly higher in Malays, descendants of natives of Sabah and Sarawak, schools in rural areas, females, and current smokers. However, age group and knowledge on the harmful effects of SHS were not significant after adjusting for confounding effects.

**CONCLUSIONS:**

A substantial proportion of school-going adolescents were exposed to secondhand smoke in the car of their parents/guardians. This highlights the need for effective tobacco control measures to include health promotion and smoke-free car regulations to be introduced to prevent severe health hazards and to reduce smoking initiation among non-smoking adolescents.

## INTRODUCTION

Secondhand smoke (SHS) is the smoke released from burning tobacco products and then exhaled by smokers^1^. It consists of gases, particulate matter, nicotine and other substances, some of which are carcinogenic^1,2^. SHS has been identified as one of the main factors of indoor air pollution^3^ for which there is no safe level of exposure^1^. One per cent of global mortality is related to SHS, with 61% of the Disability Adjusted Life Years reported among children. In addition to morbidity, SHS is estimated to cause more than 0.6 million deaths annually, 28% of which are amongst children^4^.

The higher proportion of morbidity and mortality reported among children compared to adults was due to the higher air inhalation (per body weight compared to adults), less developed immune system, and inability to move away from the source^2^. In addition, children may have a higher breathing rate. Children’s exposure to SHS increases the risk of respiratory health diseases such as coughing, asthma, bronchitis, and pneumonia, which adversely affects their lung function^5^, increases the risk of anaesthetic complications and some negative surgical outcomes in children^6^. Furthermore, exposure to SHS may affect their cognitive development^7^. The risk for non-smoking adolescents to initiate smoking is higher compared to their counterparts who are not exposed to SHS^8,9^. Therefore, the reduction of SHS exposure has been one of the main missions of the Ministry of Health, Malaysia, in order to reduce the health-related problems caused by SHS^10^.

Various studies have reported that the exposure of SHS in confined spaces such as cars results in more adverse health effects compared to exposure in other places such as home, workplace or cafe/bars, even in some public areas where smoking is allowed^11,12^. This is because the concentration of SHS is significantly higher in confined areas such as in cars^11^. Scientific evidence suggests that smoking in a car, even for a short period of time, produces a concentration of respirable particles that is potentially harmful to children^13^. In addition, the pollutants of SHS will be adsorbed onto the inside surfaces of the car and remain there for a long period, due to the types of materials used for the inside of cars^14^. Hence, there is also thirdhand smoke exposure (deposited toxicants) that further increases the health risks of passengers^15^. Studies also revealed that the source of SHS exposure in parent/guardian cars originates from parent/guardian and family members who are smoking^16^.

Studies on SHS exposure among youth and school-going adolescents have been carried out in Malaysia^17,18^. Ghazali et al.^18^ reported that more than half of the secondary school-going adolescents were exposed to SHS, and the odds of exposure were higher among male, current smokers and if one of their parents/guardians smoked. Similarly, Lim et al.^18^ who carried out a study in Peninsular Malaysia also reported similar outcomes. However, their studies measured only general exposure to SHS without elaborating on the details of the exposure. To date, only one previous study conducted in Malaysia describes SHS exposure in the car^19^, but it was only conducted in two states in Malaysia (i.e. Kedah and Melaka), and the samples were not representative of Malaysian school-going adolescents. Therefore, a suitable policy cannot be formulated to address SHS exposure effectively. Given the scarcity of data, this study aimed to assess the prevalence and factors associated with SHS exposure in parent/guardian cars among a representative sample of Malaysian school-going adolescents.

## METHODS

### Sampling and design

We conducted the Tobacco and E-Cigarette Survey among Malaysian Adolescents (TECMA) in 2016. The detail description of the study can be found in Lim et al. (2018)^20^. In brief, the TECMA study was a nationwide school-based study that employed a cross-sectional study design and multistage cluster sampling to select a representative sample of public-school-going adolescents aged 11–19 years, based on the latest sampling frame provided by the Ministry of Education, Malaysia. The first stage consisted of states in Malaysia, and the second stage comprised the division of urban and rural areas for each state. Followed by the systematic selection of the secondary or primary schools available in each state, based on proportion-to-size sampling approach, classes from the schools were selected through simple random sampling. All students from the selected classes were invited to participate in the study. In total, 138 schools were selected (82 urban and 56 rural). The sample size was determined by the estimated prevalence rate of 3%, an alpha value of 5%, a design effect of 1.5 to cater for clustering effects among students in the classes, a margin of error of 1.5%, and expected a non-response rate of 20%. Based on these parameters, 13980 respondents were required for the study. A total of 13162 adolescents participated in the study, yielding a response rate of 88.7% (n=13162/14832; estimated population 3684760/4152183).

### Questionnaire

The questionnaire was adapted from the Global Youth Tobacco Survey and then pre-validated prior to use. It was the tool used to obtain data from the selected respondents. The self-administered approach was used to collect data from the respondents, with the consent of their parents to participate in the study. The data collection was carried out in the areas provided by the school administration during the school session. The trained research team members briefed the respondents on the objective of the study. The participation in the study was based on a volunteer basis, and students had the right not to answer any of the items in the questionnaire. All information given by respondents was confidential, and their identity anonymous. In addition, the research team members also explained the items in the questionnaire and assistance was provided to respondents who needed clarification about the items. There were no school teachers or staff present during the data collection session.

The Ministry of Education and the respective Department of Education of each state approved the study, while the Ethical Committee of the Ministry of Education and the Medical Research & Ethical Committee of the Ministry of Health, Malaysia, granted ethical approval for the study. The Director-General of Health, Malaysia, granted permission for publication of this work. The authors can be contacted for data requests.

### Measurements

The dependent variable in the survey was ‘exposure to SHS’ in the car and was measured using the item: ‘During the past 7 days, have you been exposed to cigarette smoke inside your parents’/guardians’ car?’; with the choice of ‘Yes’ or ‘No’, whilst those who did not travel in their parents’/guardians’ car were instructed to skip the item during the briefing session. Those who answered ‘Yes’ were defined as being exposed to SHS in the parents’/guardians’ car. On the other hand, the independent variables in this study were: gender, age (≤12 years, 13–15 years, ≥16 years), ethnicity (Malay, Chinese, Indian, natives of Sabah and Sarawak, or other including Serani, Sikh, Siamese, Indonesia, Suluk, and foreigners), school location (urban or rural), current smoking status (Yes, smoking at least once in the last 30 days), and knowledge on the potential of SHS to be detrimental to health (No, probably No, Probably Yes, Yes).

### Data management and analysis

Data were cleaned and weighted to take into account the complex sample design and response rate to allow generalization of results to the Malaysian school-going adolescents. The characteristics of respondents were assessed through descriptive statistics. Chi-squared analyses were used to test the association between all categorical independent variables and exposure to SHS in the parents’/guardians’ car. Variables with p<0.25 in the chi-squared analysis were included in the binary multiple logistic regression to determine the influence of each variable on SHS exposure in parents’/guardians’ car. Two-way interaction analysis between the independent variables in the models was carried out. All analyses were carried out using SPSS version 20 software at an alpha level of 0.05.

## RESULTS

Among the respondents, male and female respondents were almost equally distributed (51.1% males and 48.9 % females). Two-thirds of the respondents were Malays, followed by Chinese (13.0%), and an almost equal distribution of Indian and natives of Sabah and Sarawak. Slightly more than three-quarters (76.1%) of the respondents were aged <16 years. Of the 13136 respondents (estimated population 3684760), 14.2% (524521/3684759) were current smokers (Table 1).

**Table 1:** Sociodemographic characteristics of schoolgoing adolescents that participated in the TECMA study, Malaysia 2016

*Variables*	*Estimated population*	*Sample*	*Percentage (%)*
**Gender**			
Male	1881131	6582	51.1
Female	1803629	6554	48.9
**Age** (years)			
≤12	1369393	4138	37.2
13–15	1434842	5278	38.9
≥16	880523	3720	23.9
**Ethnicity**			
Malay	2433437	9243	66.1
Chinese	477956	1764	13.0
Indian	213674	748	5.8
Native of Sabah	211781	543	5.7
Native of Sarawak	195558	447	5.7
Other	147095	385	4.0
**School area**			
Urban	1677958	7689	45.3
Rural	2006801	5448	54.5
**Current smoker**			
Yes	524231	1807	14.2
No	3160528	11329	85.8

The overall prevalence of SHS exposure in parents’/guardians’ car was 23.3%. SHS exposure was significantly higher among Malay (26.4%, 95% CI: 24.9–27.9%), native of Sabah (24.6%, 95% CI: 20.4–29.3%), and native of Sarawak school-going adolescents (27.3%, 95% CI: 23.0–32.3%) compared to the Chinese students (11.9%, 95% CI: 9.9–14.1%), current smokers (35.1%, 95% CI: 31.2–39.1%) and schools in rural areas (26.9%, 95 CI: 25.2–28.7%). A similar observation was identified in the multivariable analysis, in which the likelihood of SHS exposure was significantly higher among Malays, natives of Sabah and Sarawak, current smokers, and females. However, knowledge of health effects of SHS, which was significant in univariate analysis (16.6% thought that SHS was not harmful to health) was not significant after controlling for the effects of other independent variables (Table 2).

**Table 2:** Prevalence of SHS exposure inside the car of their parents/guardians and multivariable logistic regression to determine the associated factors, Malaysia 2016

*Variables*	*Estimated population*	*Sample*	*% (95% CI)*	*P*	*AOR (95% CI)*
**Overall**	857360	2849	23.3 (22.2–24.4)		
**Gender**					
Male	423825	1403	22.5 (21.1–24.0)	0.177	0.83 (0.71–0.98)
Female	433534	1446	24.1 (22.4–25.8)		Ref.
**Age** (years)					
≤12	281312	851	20.6 (19.1–22.2)	0.001	Ref.
13-15	362213	1202	24.2 (23.3–26.4)		1.17 (0.89–1.54)
≥16	213834	796	24.3 (22.1–26.6)		1.15 (0.88–1.50)
**Ethnicity**					
Malay	641373	2263	26.4 (24.9–27.9)	<0.001	2.34 (1.64–3.08)
Chinese	56444	189	11.8 (9.9–14.1)		Ref.
Indian	27144	84	12.7 (9.7–16.5)		1.03 (0.65–1.65)
Native of Sabah	51916	130	24.6 (20.4–29.3)		1.83 (1.32–2.56)
Native of Sarawak	54402	111	27.3 (23.0–32.3)		2.26 (1.50–3.41)
Other	25303	10	17.3 (13.1–22.4)		1.40 (0.86–2.26)
**School area**					
Urban	317217	1475	18.9 (17.7–20.2)	<0.001	Ref.
Rural	540142	1374	26.9 (25.2–28.7)		1.32 (1.03–1.67)
**Current smoker**					
Yes	144741	429	35.1 (31.2–39.1)	<0.001	1.85 (1.60–2.14)
No	662303	2256	21.2 (20.1–22.3)		Ref.
**Perceived SHS as harmful to health**					
No	36673	115	16.6 (12.4–21.9)	0.001	0.79 (0.58–1.07)
Probably No	29807	99	18.6 (14.5–23.6)		0.84 (0.63–1.12)
Probably Yes	208784	643	25.6 (22.5–29.1)		1.07 (0.94–1.23)
Yes	581785	1990	23.4 (20.4–26.7)		1

AOR: adjusted odds ratio. CI: confidence interval.

## DISCUSSION

The SHS exposure rate of 23.3% in this study is very similar to the findings by Azagba et al.^21^ in Canada, to the 21.4% exposure rate reported by Agaku et al.^22^ among middle school students in the USA and the value of 23.0% reported among youth in New Zealand^23^. The prevalence in this study is slightly higher than the exposure rates of 19%^24^ and 17.3%^19^ reported among youth in England and Malaysia, respectively. However, the prevalence rate is lower than reported by studies conducted in Portugal and South Carolina, USA, respectively^16,25^. The differences of sociodemographic characteristics between the respondents in our study and those of Abidin et al. might explain the different prevalence rates between both studies^19^. The findings are very encouraging, in view of the fact that legislation of smoking prohibition in private vehicles exists in Canada and New Zealand^21,23^, whereas no such provision exists in Malaysia but the rate of SHS exposure is similar. However, continual concerted efforts should be carried out to reduce and eliminate SHS exposure among youth in vehicles to the level (9%) that has been reported, for instance, in Wales^26^.

The likelihood of SHS exposure in the parents’/guardians’ car was significantly higher among female respondents. This finding was unexpected in view of the lower prevalence of smoking among female adults or female adolescents in Malaysia. However, the finding is consistent with the outcomes of the studies conducted by Azagba et al.^21^ and Agaku et al.^22^ among school-going adolescents in Canada and the USA, respectively. We postulate that the source of SHS exposure could mostly originate from their parents/guardians (mostly father) in view of the higher smoking prevalence of male adults in Malaysia. Malaysia is a patriarchal society (i.e. a social system where the father is the head of the family), and the young have restricted autonomy. The sense of respect of the young for their elders discourages them from complaining about smoking in the car of the elders, especially among their fathers. Therefore, they are less likely to dissuade their parents/guardians from smoking in the car. This postulation was strengthened by the better knowledge and awareness of SHS’s harmful effects among female students compared to males, and there is no significant association between the level of knowledge of the harmful effects of SHS among respondents and exposure to SHS in their parents’/guardians’ car. Another plausible reason might be that parents/guardians in Malaysia are more protective of females compared to males. This partly explains why gender-specific car exposure depends on the socio-cultural environment. As a result, it is suggested that Malaysian female students are more exposed to SHS in their parents’/guardians’ cars because they have a closer relationship with their fathers. However, the hypothesis should be tested in the future through in-depth qualitative and quantitative studies to determine the parents’/guardians’ relationship with their child according to gender.

Exposure to SHS was significantly higher among smokers. We postulated that SHS exposure in the parents’/guardians’ car mostly originates from parents/guardians, family members or respondents who smoke, who may be more lenient towards smoking in their car and are unlikely to prohibit guests or household members, or their children, from smoking inside the vehicle^17,18,27^. No positive relationship was observed between age and exposure to SHS in parents’/guardians’ cars, after adjusting for the confounding effect of other independent variables. This is in line with other studies among middle and high school USA students^22^, however differs from studies in Germany where parents/guardians are more concerned about the younger teenagers’ health and safety, and thus exert more control and limit their sons’/daughters’ exposure to the health hazards of SHS^28^. This calls for in-depth qualitative studies among the parents/guardians to be carried out to elucidate the factors contributing to the findings in this study.

Higher odds of SHS exposure in parents’/guardians’ cars were reported among respondents from schools in rural areas, among the Malay and natives of Sabah and Sarawak compared to their counterparts in urban areas, Chinese and Indian descendants. The higher prevalence of smoking among adult Malays and natives of Sabah and Sarawak, and those who reside in rural areas^29^, might indicate that smoking is a norm in these groups and, therefore, smoking in any locality, including in the parents’/guardians’ car, is accepted by society. This phenomenon might increase the risk of exposure to SHS among adolescents from schools in rural areas and those of Malay, and native of Sabah and Sarawak, descent. Our postulation is supported by the findings that a higher SHS exposure at home was also reported among similar ethnic groups and residents in rural areas^29^. However, more studies are required to investigate the actual factors associated with higher SHS exposure in parents’/guardians’ cars, including smoking restrictions, cultural aspects and social norms of smoking among different ethnicities in Malaysia.

### Strengths and limitations

This study was not without limitations. Exposure to SHS in parents’/guardians’ cars was self-reported and was not verified through objective measurements and biomarkers (i.e. serum cotinine); therefore, this might introduce a recall bias of SHS exposure. We also did not measure the frequency of SHS exposure in our study, although there is no safe level of SHS exposure^16^, many studies have shown a dose-response relationship between SHS and adverse health effects^30,31^. In addition, independent variables that have been shown to be significant in other studies, such as other risky behaviours (such as alcohol consumption)^21^, smoking restrictions in the car^32^ and smoking status of respondents’ family members, were not accounted for in this study^16^. Furthermore, the detailed sociodemographic background of respondents (e.g. income level of respondents’ parents/guardians) was not measured in this study. However, the representativeness and large sample size and high response rate among respondents, enable the generalization of the results to the school-going adolescent population in Malaysia.

## CONCLUSIONS

These results highlight that Malaysian school-going adolescents are frequently exposed to SHS in their parents’/guardians’ car. However, the vast majority of youth are aware of the harmful effects of SHS. Considering the health and social consequences associated with SHS exposure, tailored public health policies and measures such as health promotion activities to increase awareness on the adverse health hazards of smoking among parents/guardians are necessary. In addition, parents/guardians who smoke should be advised to quit and discourage others from smoking in their car. These measures should be given priority since youth lack the ability to avoid SHS in their parents’/guardians’ car, which is exacerbated by the community culture that limits the rights of children compared with those of adults. In addition, the implementation of the KOSPEN (*Komuniti Sihat, Pembina Negara* – Healthy Community, Nation Builder) community intervention programme by the Ministry of Health of Malaysia should be expanded to include the theme of cigarette smoke-free cars, besides the advocacy of cigarette smoke-free homes^33^. Furthermore, the introduction of a cigarette smoke-free regulation in cars may be the way forward to reduce SHS exposure in view of the efficacy of the regulations implemented elsewhere^25,34^ and since the majority of Malaysian adults and youth support smoke-free initiatives^35,36^.
